# Monitoring Endangered and Rare Wildlife in the Field: A Foundation Deep Learning Model Integrating Human Knowledge for Incremental Recognition with Few Data and Low Cost

**DOI:** 10.3390/ani13203168

**Published:** 2023-10-10

**Authors:** Chao Mou, Aokang Liang, Chunying Hu, Fanyu Meng, Baixun Han, Fu Xu

**Affiliations:** 1 School of Information Science and Technology, Beijing Forestry University, Beijing 100083, China; chao_m@bjfu.edu.cn (C.M.);; 2 Engineering Research Center for Forestry-oriented Intelligent Information Processing of National Forestry and Grassland Administration, Beijing 100083, China; 3 State Key Laboratory of Efficient Production of Forest Resources, Beijing 100083, China

**Keywords:** endangered and rare wildlife monitoring, foundation deep learning model, few-shot task, human knowledge fusion, incremental animal monitoring

## Abstract

**Simple Summary:**

Intelligent monitoring of endangered and rare wildlife using deep learning is important for biodiversity conservation. The present study aims to train deep learning recognition models using the limited animal samples and computational resources available in real-world field monitoring scenarios. Inspired by the ability of human zoologists to quickly identify endangered and rare wildlife species based on only one single picture, we incorporate expert knowledge to create an excellent recognition model with these practical constraints. The empirical evidence discovered in this study indicates that the insightful incorporation of specialist knowledge can meaningfully improve an algorithm’s accuracy, even with limited sample sizes. Additionally, our experimental results validate the usefulness and efficacy of our discoveries in identifying previously unknown species. This work presents the first exploration of a practical solution for endangered and rare wildlife monitoring using a foundational deep learning model. Automated monitoring capability for unidentified species has the potential to facilitate breakthroughs in the fields of zoology and biodiversity research.

**Abstract:**

Intelligent monitoring of endangered and rare wildlife is important for biodiversity conservation. In practical monitoring, few animal data are available to train recognition algorithms. The system must, therefore, achieve high accuracy with limited resources. Simultaneously, zoologists expect the system to be able to discover unknown species to make significant discoveries. To date, none of the current algorithms have these abilities. Therefore, this paper proposed a KI-CLIP method. Firstly, by first introducing CLIP, a foundation deep learning model that has not yet been applied in animal fields, the powerful recognition capability with few training resources is exploited with an additional shallow network. Secondly, inspired by the single-image recognition abilities of zoologists, we incorporate easily accessible expert description texts to improve performance with few samples. Finally, a simple incremental learning module is designed to detect unknown species. We conducted extensive comparative experiments, ablation experiments, and case studies on 12 datasets containing real data. The results validate the effectiveness of KI-CLIP, which can be trained on multiple real scenarios in seconds, achieving in our study over 90% recognition accuracy with only 8 training samples, and over 97% with 16 training samples. In conclusion, KI-CLIP is suitable for practical animal monitoring.

## 1. Introduction

The conservation of endangered and rare wildlife is a primary part of preserving biodiversity [1,2]. Researchers and wildlife management agencies in many countries are monitoring animals in the wild for conservation purposes as animal diversity is declining at an unprecedented rate [3,4]. Conventionally, animal monitoring is studied by human experts who gather data through surveys [5,6,7], tracking [8,9,10,11], sampling [12,13,14,15], and other observational ways [16,17]. However, there are significant labor and time costs associated with these traditional methods [18]. Meanwhile, these traditional methods are also prone to data bias due to animal disturbance and observer subjectivity, which are often unavoidable [19,20]. To tackle these issues, the new generation of information technology is being used to monitor animals intelligently [3]. Firstly, inexpensive and accessible sensing devices (e.g., camera traps [21], consumer cameras [22], and acoustic sensors [23]) are being deployed in the field to accelerate data acquisition in a cost-effective and objective manner [3]. Then, intelligent algorithms such as deep learning models are employed to automate the monitoring of wildlife species by analyzing the massive amount of data collected [3], as they have been successfully applied in many fields [24,25,26].

Recently, and not surprisingly, it has become increasingly popular to use next-generation information technology to monitor rare and endangered wildlife [3,27,28,29]. For instance, China has established the national intelligent wildlife monitoring system (including camera traps and deep learning models) in a number of national parks, such as the “Giant Panda National Park” and the “Northeast China Tiger and Leopard National Park” [27,28]. In general, the proficiency of monitoring is mainly dependent on the effectiveness of intelligent algorithms. Therefore, deep learning models with high recognition performance, such as convolutional neural networks, have recently received significant attention in academic research [28,29,30]. In fact, large deep learning models, also known as foundation deep learning models, such as CLIP [31] and chatGPT [32], have demonstrated exceptional performance in industry fields. However, the field of wildlife monitoring has not yet benefited from the power of foundation deep learning models. As foundation deep learning models are much more powerful than currently used neural networks [31,32], animal ecology researchers can monitor wildlife more accurately, efficiently, and effectively by introducing these models. Particularly as species-level wildlife classification of endangered and rare wildlife is an important basic task in intelligent wildlife monitoring [3], we are motivated to investigate the recognition of endangered and rare wildlife species through the use of foundational deep learning models for automated animal monitoring.

As is widely acknowledged, big data plays a crucial role in the success of foundational deep learning models [32,33]. At first glance, the use of large sensing devices may seem beneficial, as they can collect a wealth of surveillance image data. However, issues surrounding the availability and quality of data still pose a significant obstacle to the application of foundational deep learning models in wildlife monitoring, particularly for endangered and rare species [3]. On one hand, even with numerous sensors deployed over a vast area, obtaining a significant amount of usable data is challenging due to environmental and meteorological disturbances, animal behavior, and other issues [34,35]. On the other hand, collecting data on small numbers of endangered and rare wildlife individuals like pandas can be likened to searching for a needle in a haystack. Moreover, wildlife conservation data are often not publicly available and the massive labeled data required to train the deep learning model is extremely costly [3]. The lack of access to available and labeled big data makes it impossible to fine-tune [36] a foundational model, which is the conventional approach used for training a deep leaning model for recognizing wildlife [30]. Furthermore, the application of foundation deep learning models in field monitoring situations encounters obstacles like financial and ecological problems stemming from the need for massive amounts of training data [3] and deployment on portable devices with low computational power [37,38]. Thus, we aim to apply foundation deep learning models to animal ecology with limited image data and low computational cost in this study.

In computer science, the challenge of training large models with limited labeled data is referred to as the *few-shot learning* problem [39]. Three common approaches to learn from small datasets include fine-tuning models, data augmentation, and transfer learning [40]. However, all of these techniques still require a minimum amount of labeled data and computational resources [30,41]. Due to the difficulty in obtaining data on endangered and rare wildlife [3], and the limited computing power available in the field environment [37], existing foundation training methods are not applicable to monitoring endangered and rare wildlife. Thus, to improve the performance of foundation models with fewer samples we draw inspiration from the ability of human zoologists to accurately identify animals from just a few animal images, and explore ways to integrate their expert knowledge. At the same time, computer science research confirms the effects of introducing more information in a similar way [42]. Nevertheless, the question of how to integrate domain knowledge into a foundational deep learning model for few-shot learning with low computational cost is challenging. It is worth noting that distinguishing between different animal species typically involves describing morphological characteristics, habits, and other factors [43]. In our study, we concentrate on the impartial depictions of fauna by experts. This approach provides the benefit that, while obtaining images of at-risk species can be difficult, written descriptions are easily accessible. In addition, with the multitude of large language models (one type of foundation deep learning models) currently available, sophisticated techniques can be employed to use expert knowledge from computer science [44]. As a result, the CLIP model [31] has been selected for its ability to efficiently and effectively fuse textual knowledge and images through a simple point product similarity for few-shot learning. Specifically, our approach incorporates a lightweight adaptive network that eliminates the need to train CLIP, resulting in significantly reduced training costs.

When monitoring animals in real-life situations, there may be challenging cases where previously unidentified species are encountered. This can either involve species that have never been observed or newly discovered ones. The former is known as “zero-shot learning” and is the most rigorous form of few-shot learning as it involves deep learning models encountering images of species that they have not seen before. The latter involves monitoring newly discovered species. In practical scenarios zoologists expect the system to be able to discover unknown species to make significant discoveries. For example, Wuyi Mountain National Park in China has used the monitoring system to discover 17 new species in the past eight years (available online: http://www.forestry.gov.cn/lyj/1/lcdt/20230821/518056.html, accessed on 22 August 2023). However, the identification of new species mainly depends on human review of surveillance videos that the current system cannot accurately recognize. What can be expected is that equipping the system with an automated monitoring capability for unidentified species holds immense potential for breakthroughs in the fields of zoological disciplines and biodiversity research. Thus, the purpose of the present work is to explore novel intelligent methods that can automatically and incrementally monitor wildlife, given practical constraints such as limited data and computational power.

## 2. Related Work

### 2.1. Intelligent Wildlife Monitoring

Many traditional wild animal monitoring methods have been widely used [16,17], including surveys [5,6,7], tracking [8,9,10,11], sampling [12,13,14,15], and so on. All of these methods are labor intensive, especially for investigating rare wild animals such as giant pandas [45]. Fortunately, with the development of information technology and artificial intelligence (AI), ground remote sensing devices (such as ground trap cameras [21]) have begun to be applied to automatically monitor wildlife [46]. For instance, Chen et al. [47] used a deep convolutional neural network to recognize species from images captured by trap cameras for wild animal monitoring. Zualkernan et al. [38] used the Xception model (a classical deep learning model) to recognize wildlife images captured by camera traps. Therefore, deep learning technology advances wildlife monitoring towards greater automation and intelligence due to its high accuracy in the recognition of wildlife. For instance, Villa et al. [48] evaluated some classical deep convolutional neural network frameworks such as AlexNet, VGGNet, and GoogleNet for identifying species. They claimed that the recognition accuracy of those traditional deep learning models could achieved 88.9% when using a high-quality dataset. As a result, zoologists and government agencies have developed intelligent systems for monitoring wildlife to enhance the efficiency and effectiveness of ecological diversity conservation [27,28]. For example, China has implemented intelligent monitoring systems by using camera traps and deep learning models to automate wildlife monitoring in several national parks such as *Giant Panda National Park* and *Northeast Tiger and Leopard National Park* [27,28].

However, current intelligent wildlife monitoring systems remain highly ineffective in monitoring small populations of rare and endangered wildlife and even species yet to be identified. The reason is that deep learning models used in these systems are hindered by data issues. Data collected by sensing devices can be noisy, fuzzy, and blurred due to changing environmental and meteorological conditions, leading to a reduction in the available data [49,50]. Obtaining high-quality data on endangered and rare wildlife is more challenging, since they are less numerous and less likely to be photographed. At the same time, deep learning models usually are challenged by inferior quality data. For example, in the work of Villa et al. [48] the accuracy of deep learning models drops dramatically, to 35.4%, when meeting unbalanced data. On the other hand, obtaining labeled training data for deep learning models often requires a significant investment due to the vast quantities of data gathered by camera traps. For instance, annotating one million images requires a trained expert to spend approximately 200 working days [50]. In a word, the limited data available for endangered and rare wildlife results in underperforming monitoring systems. Furthermore, the insufficient data results in the ineffectiveness of deep learning models for unknown species. Solving the issue of monitoring endangered and rare wildlife using deep learning methods has the potential to significantly aid in the preservation of biodiversity.

### 2.2. Deep Learning Technology

Deep learning models typically comprise multi-layer neural networks that can extract intricate patterns from vast amounts of data [51]. The primary use of intelligent wildlife monitoring lies in the classification capability of deep learning for specific species recognition [28,29,30]. Therefore, we need to obtain a large amount of high-quality, labeled data to train high-precision deep learning models to accurately monitor wildlife due to the requirements of the classification task [48]. In addition to the data problem, the problem of computational cost needs to be solved if we want to use deep learning methods to monitor endangered and rare wildlife in practice. For example, Zualkernan et al. [38] utilized deep learning on edge devices to provide real-time animal sighting data from live camera shots. However, existing deep learning algorithms are expensive to train, often requiring a week or even months of training on a computer with eight computing cards [52]. Hence, designing a lightweight deep neural network that can accomplish model training with less computation is a good idea for monitoring wildlife in the field [29]. In general, reducing the size of the model corresponds to a decrease in performance [51]. Maintaining accuracy while creating a lightweight network poses a significant challenge [29].

In addition, zoologists are very concerned about unknown species in any monitoring scenarios [53]. Unfortunately, deep learning models are weak at identifying samples (i.e., animals in the field) which they have not yet seen in the training procedure [51]. This is also defined as *incremental learning* in computer science, which refers to the ability of a machine learning system to retain the performance of an old task on the new task [54]. For example, a face recognition system should be able to add new persons without forgetting the faces already learned [54]. However, there are few studies that focus on incremental intelligent wildlife monitoring algorithms and the state-of-the-art accuracy currently stands at 77.09% [55]. Clearly this is not at a level that can be applied in practice. Hence, in this study, we pursue incremental learning capabilities for the identification of unmonitored and unknown species with high accuracy.

### 2.3. Few-Shot Leaning

As a simple introduction, in Section 1, *few-shot learning* refers to training a deep learning model with limited data [39]. It is also called the X-shot task, where the ’X’ is the number of training samples [31]. If x=0, none of training samples are used and that process is known as *zero-shot learning*. Hence, *zero-shot learning* is the most rigorous situation of *few-shot learning*. There are three common approaches to help deep learning models learn from limited data: fine-tuning models, data augmentation, and transfer learning [40]. Fine-tuning is one of the most commonly used methods to solve the few-shot problem, by training deep neural networks to learn generalized features on large-scale datasets and fine-tuning the parameters on a small number of task samples [56,57]. For example, in industry, fine-tuning can be used to detect equipment wear using a small number of ferrographic images [56]. Data augmentation resolves the issue of inadequate samples through a different approach of creating new data samples by applying a variety of transformations to existing samples [58,59,60]. For instance, Hu et al. [60] developed an adaptive convolutional neural network for diagnosing faults in industrial settings via a data simulation approach. Transfer learning is a machine learning approach that uses models trained on the source domain or task to aid in the learning process of the target domain or task [61,62,63]. For instance, Rostami et al. [63] achieved successful classification and detection of SAR data with limited labeled data by transferring a high-performance electro-optical domain model. It should be noted that there is still a need for a certain amount of labeled data and computational resources to train deep learning models [30,41]. Obviously these methods are not suitable for monitoring endangered and rare wildlife due to the minimal data and computational power issues, thereby creating a significant challenge. This challenge was the impetus for the research reported in this paper.

Moreover, in computer science there are some benchmark datasets, that are free to use, that can be used to validate the performance of models on *few-shot learning* tasks or even *zero-shot learning* tasks [64,65,66,67,68,69,70,71,72]. The 11 public benchmark datasets we used in this study are *Caltech101* [64], *OxfordPets* [65], *StanfordCars* [66], *Flowers102* [67], *Food101* [68], *FGVCAircraft* [69], *DTD* [70], *EuroSAT* [71], *CUB-200-2011* [72], and *AP-10K* [73]. Due to the lack of publicly available datasets on rare and endangered wildlife, we obtained additional data by deploying camera traps and field surveys.

It is worthy to note that the evaluation metric used in computer science for *few-shot learning* or *zero-shot learning* is the *accuracy* [31,39]. In most cases, the *precision*, *recall*, or *F1 score*, used to measure the performance of algorithms because of the unbalanced problem, are not required for few-shot or zero-shot learning because there are fewer training samples in each category [51]. In this work, we also used the accuracy as the evaluation metric.

### 2.4. Foundation Deep Learning Models

Foundation deep learning models refer to large deep learning models such as chatGPT, CLIP, etc., that have performed far beyond the classical convolutional neural networks in various tasks due to their superior modeling capabilities [74,75]. For instance, chatGPT utilizes mega-data and computational resources to generate human-like responses and has been used in the healthcare industry [76]. PointCLIP combines 3D data with training language knowledge through CLIP, enabling powerful open-world 3D learning capabilities that are not possible with convolutional neural networks [77]. However, following an extensive literature search, we did not find any studies that use foundation deep learning models for endangered and rare wildlife monitoring. Therefore, we focused on how large models can be utilized to provide enhanced monitoring capabilities for zoologists.

**Figure 1:**
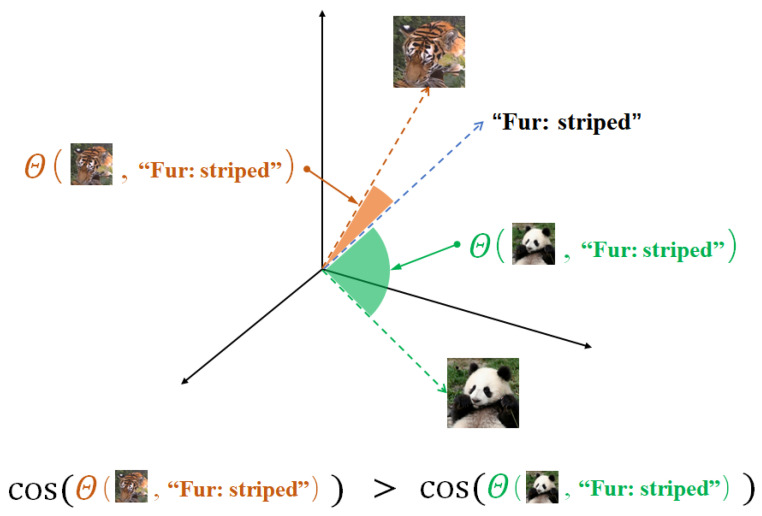
The example of dot product similarity using CLIP.

Furthermore, inspired by human taxonomists’ efficient identification of wildlife species from individual images, this paper integrates textual expert knowledge to address the few-shot problem (mentioned in Section 1). As different textual and visual modalities of knowledge need to be integrated while feeding to the classification models, we choose the vision–language model CLIP in this work. CLIP is a large pre-trained model consisting of a visual coder and a text encoder [31]. Different data types are encoded by the encoder of each corresponding modality and then connected through a simple *cosine* dot product calculation to establish a similarity relationship [31]. In particular, CLIP is obtained by pre-training on 400 million “image–text pairs”. This means that, on one hand, the CLIP model contains a huge amount of knowledge and, on the other hand, it becomes almost impossible to fine-tune it directly with a small number of samples. Therefore, we fixed CLIP and fine-tuned an additional lightweight network to use it. This incorporates text-based expert knowledge and also utilizes the large amount of prior knowledge embedded in CLIP. As shown in Figure 1, the trained CLIP model can help us calculate the similarity scores between text-based expert knowledge (e.g., fur:striped) and the image samples of the test species, which can help us recognize the species. Crucially, it is also possible to adapt CLIP to real-world scenarios of endangered and rare wildlife monitoring by training this extra network with fewer samples and lower computational cost. The details will be introduced in the following sections.

## 3. Materials and Methods

### 3.1. Datasets

As shown in Table 1, a total of 12 datasets covering three main categories (i.e., general data, animal data, and endangered and rare wildlife data) are tested in this work. Firstly, 8 public image datasets (i.e., *No. 1*∼*No. 8* as shown in Table 1) which are commonly used to test performance, including few-shot learning of CLIP and its derived models such as ours, are selected. Secondly, four animal datasets (i.e., *No. 9*∼*No. 12* as shown in Table 1) are selected to test of the performance of our method in animal classification. The *No. 9* dataset (*CUB-200-2011*) is a bird-related benchmark dataset, which is also often used to test few-shot learning and animal detection algorithms [78]. Due to the low probability of the occurrence of unknown species, we chose a dataset with fewer species to validate the incremental learning. So, a dataset consisting of 10% of the species from the *No. 9* dataset was randomly chosen to form the *No. 10* dataset (*CUB-20-2013*). Due to the lack of animal benchmark datasets, data on animal species of 50 classes from AP-10K and ATRW were selected to form the *No. 11* dataset (*Animals*). Finally, the *No. 12* dataset consists of 10 endangered and rare wildlife species actually captured in the field by trap cameras in China’s national parks. A large number of datasets of different types are sufficient to illustrate the effectiveness of the proposed KI-CLIP method.

In more detail, datasets *No. 1* to *No. 9* are benchmark datasets commonly used for validating few-shot and zero-shot tasks in computer science, and they can be freely obtained through references [64,65,66,67,68,69,70,71,72], respectively. Their categories comprise wildlife, livestock, pets, birds, plants, food, and numerous other common groups in daily life. Hence, the variety of categories provides diverse perspectives for showcasing the competence of intelligent algorithms. Moreover, the foundational benchmark datasets (i.e., *CUB-20-2013*, *AP-10K*, and *ATRW*) about animals from which the *No. 10* and *No. 11* datasets were extracted can also be downloaded for free from the URLs provided in [72,73,79]. Ten endangered and rare species of wildlife captured by trap cameras we deployed in the field or photographed during field surveys over the past three years were chosen to form the *No. 12* dataset. Since only the *No. 10*, *No. 11*, and *No. 12* datasets are non-benchmark datasets, their details and examples are shown in Table 2.

### 3.2. KI-CLIP

#### 3.2.1. The Framework of KI-CLIP

**Figure 2:**
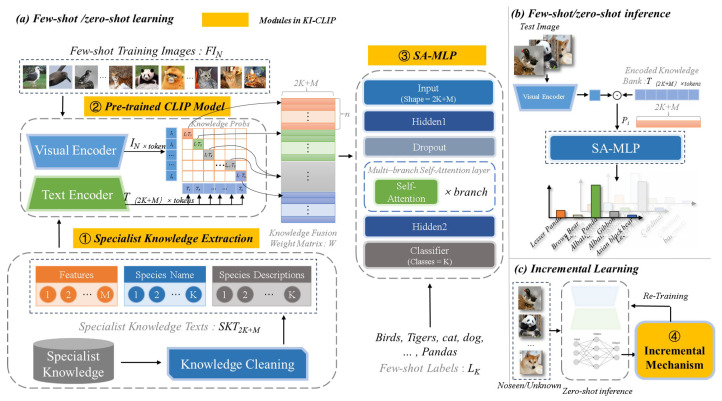
The description of KI-CLIP and its application to the recognition of endangered and rare wildlife in practice. (**a**) Description of few-shot or even zero-shot learning in KI-CLIP. (**b**) Description of the usage of KI-CLIP in the field. (**c**) Description of incremental learning in KI-CLIP.

In this study, we proposed the so-called **KI-CLIP** method for incremental recognition of endangered and rare wildlife with little data and low cost by integrating textual expert **K**nowledge with **I**mage data based on **CLIP**. For the purpose of accurate few-shot learning, the powerful pre-trained CLIP model is used to fuse textual human expert knowledge and image data for wildlife recognition. It is worth noting that the CLIP model is used in inference mode without any training consumption. In addition, a light multilayer perceptron (MLP) with four weight layers is designed to use the fusion features for animal identification. And several low-cost attention branches are embedded in MLP to improve the capability of the model [80]. We also added a simple residual structure and a dropout layer to avoid overfitting [81]. In short, training with little data and computational resources is possible due to the lightweight structural design [82]. In addition, a simple incremental learning structure based on predicted probabilities is designed with the computational cost constraint of throwing. Figure 2 shows the proposed KI-CLIP and its application to the recognition of endangered and rare wildlife in practice.

As shown in Figure 2, the proposed KI-CLIP consists of four modules: *specialist knowledge extraction (#1)*, *pre-trained CLIP model (#2)*, *SA-MLP* (short for self-attention MLP, #3), and *incremental mechanism (#4)*, as shown in Figure 2. In KI-CLIP, for the purpose of few-shot learning (mentioned in Section 1), human expert knowledge is vectorized by the *#1 Specialist knowledge extraction* module to establish the correlation with the training image data in a *#2 pre-trained CLIP model*. Subsequently, a lightweight shallow *#3 SA-MLP* network with a self-learning mechanism is then trained to enable realistic applications. Due to the minimal data and computational resources used it can be rapidly retrained, determined by a probability-based *#4 incremental mechanism* module. In summary, the few-shot (even zero-shot) and incremental inference capabilities make KI-CLIP capable of recognizing unseen and unknown endangered and valuable wildlife in the field.

*Specialist Knowledge Extraction (#1 module)*. The textual specialist knowledge is extracted automatically from web sources (e.g., digital journal articles and textbooks), so that the amount of knowledge about the species can be quickly collected in practice. These raw data are then cleaned to the *specialist knowledge texts* form, as shown in Table 3. As shown in Table 3, the *specialist knowledge texts* has three main files including features, species name, and species description. Then, each input specialist knowledge text data $SKT_{2K+M}$ of KI-CLIP is concatenated by these fields using Equation (1).

(1) $$SKT_{\left(2K+M\right)}=⊕\left(F_{M},S_{K},D_{K}\right),$$

where ⊕(·) is the concat function, $F_{M}$ is the total features in KI-CLIP, $S_{K}$ is the total species names in KI-CLIP, and $D_{K}$ is the total description in KI-CLIP.

In particular, in this cleaning process, the open source large language model *Llama 2* [83] has been used to improve the cleaning efficiency, and it has been confirmed by human domain experts. In real-world wildlife monitoring scenarios, human zoologists are always the best “models” [84], and unknown species need to be processed by zoologists. It is, therefore, reasonable to involve zoologists in the process of knowledge cleaning. In contrast, the short and concise knowledge text could be easily collected by human experts. The knowledge text is suitable for real application.

*Pre-trained CLIP Model (#2 module)*. On the one hand, the CLIP model in KI-CLIP is designed to fuse data from different modalities of images and specialist knowledge text. On the other hand, the few-shot or even zero-shot capability from CLIP in KI-CLIP is useful in real-world intelligent wildlife monitoring. Once the pre-trained CLIP model is determined, the text encoding matrix $T_{(2K+M)\times tokens}$ (also called encoded knowledge bank) and visual encoding matrix $I_{N\times tokens}$ are obtained after passing the specialist knowledge text data $SKT_{\left(2K+M\right)}$ and *N* few-shot training images $FI_{N}$ through the text encoder and visual encoder of CLIP, respectively. The size of the tokens are determined by the patch size set in CLIP, and *N* image samples include *K* classes with *n* samples in each class. Finally, the fusion knowledge weights $W_{N\times (2K+M)}$ are obtained by a simple similarity calculation in CLIP to establish a link between images and expert knowledge of animal species. As only the matrix *W* is used as input for subsequent training, the pre-trained CLIP has exactly zero training consumption. This exploits both the power of large foundation deep learning model under constrained computational resources and the few-shot learning ability to incorporate expert knowledge to meet real-world animal monitoring requirements. The calculation can be found from Equations (2)–(4).

(2) $$T_{(2K+M)\times tokens}=Text\_Encoder\left(SKT_{\left(2K+M\right)}\right),$$

(3) $$I_{N\times tokens}=Visual\_Encoder\left(FI_{N}\right),$$

(4) $$W_{N\times (2K+M)}=I_{N\times tokens}\cdot T_{(2K+M)\times tokens},$$

where tokens is the embedding size determined by the patch size in CLIP, *N* is the total number of few-shot training wildlife images, and N=n×K, with *n* being the number of samples in each class.

**Figure 3:**
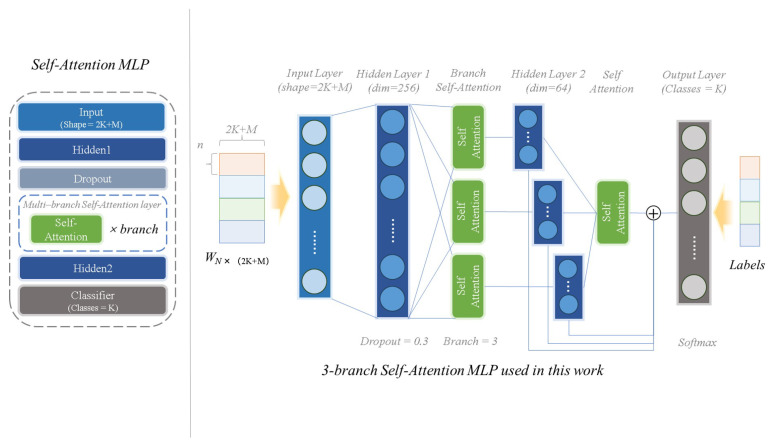
The illustration of SA-MLP used in this study.

*SA-MLP (#3 module)*. As illustrated in Figure 3, SA-MLP is a multi-branch shallow fully connected neural network. There are only two hidden layers and three branch self-attention layers. A shallower network means that less training data and computational resources are required. Meanwhile, wider networks lead to better performance. Combining the less computationally intensive nature of self-attention and the ability to capture context, the multiple branches of the self-attention layer are then designed into the MLP in order to better learn the complex mapping relationships in the shallow network. Considering that there are three fields (see Table 1) in the expert knowledge texts, the number of branches is set to three in this work. Furthermore, to avoid overfitting, the residual structure and dropout layers are also embedded. An additional self-attention layer is used in the dropout layer and residual structure to reduce computation. To summarize, SA-MLP benefits from a light structure, requires little training data, and is computationally inexpensive. As the *#2 module* (pre-trained CLIP) is used without training, KI-CLIP also requires minimal resources.

*Incremental Mechanism (#4 module).* In order to deal with the problem of unseen and unknown species often encountered in real-world monitoring of endangered and rare wildlife, we design an incremental learning mechanism based on the determination of the classification probability distribution $f_{softmax}$ in softmax. As shown in Equation (5), the incremental learning module is a binary function. Incremental learning is activated when I=1, i.e., KI-CLIP is retrained. In particular, because the pre-trained CLIP is frozen, we only have to use a smaller amount of resources to retrain the SA-MLP. For unseen endangered and rare wildlife, retraining only occurs if zero-shot inference fails. This also means that if the zero-shot ability of KI-CLIP works well, the incremental learning does not need to be activated all the time. For unknown endangered and rare wildlife, as the specialist knowledge is blank, we need to interact with human experts when retraining KI-CLIP. Zoologists can also benefit from this process.

(5) $$I=\left\{\begin{array}{cc} 1, & E\left(f_{softmax}\right)\in \left[\mu -\gamma ,\mu +\gamma \right],\\ 0, & else \end{array}\right.$$

where E(·) is the expected function, μ is the expected value of probability distribution U∼(0,K), and γ is the fixed or learnable tolerance factor, which is fixed to (1+0.2)/K.

#### 3.2.2. Application of KI-CLIP in Real-Life Scenarios

The use of KI-CLIP for the identification of endangered and rare animals in real-life scenarios consists of the following three steps: first, a few-shot or even zero-shot learning is carried out to obtain a deep learning model that can be used; then, this model is used to infer recognition from photographed animals; finally, the model is retrained to improve its recognition performance if the incremental learning module finds unseen animals or unknown species. These stages are paired with the three parts, *(a) few-shot/zero-shot learning*, *(b) few-shot/zero-shot inference*, and *(c) incremental learning*, of Figure 2, which we will describe in the following section.

*Few-shot/zero-shot learning.* First, the specialist knowledge regarding the monitored endangered and rare wildlife is extracted from Wikipedia and human experts. Then, the human expert knowledge, the few image data, and annotated information about the species are used to train the KI-CLIP model with few-shot status. The prior knowledge of the fixed *pre-trained CLIP (#2 module)* is used to solve the training with limited data and significantly reduce the computational resources. Specifically, species that have never been photographed in the monitoring area (i.e., the unseen species) require only their expert knowledge for training, also known as zero-shot learning. As a result, the knowledge fusion weight matrix *W* is obtained. Next, the *SA-MLP (#3 module)* is trained by using *W* with the loss function, as in Equation (6). Therefore, the few-shot or even zero-shot learning capability of KI-CLIP is driven by expert knowledge and the CLIP model, and it is well suited to endangered and valuable wildlife with very small numbers and images. In addition, the minimal computational cost of training allows for the following incremental learning through retraining with few or even zero samples.

(6) $$L\left(out,label\right)=-\log\frac{e^{x_{N\times K}^{label}}}{\sum_{i=1}^{N}e^{W_{1\times (2K+M)}^{i}}},$$

where L(·) is the loss function and $X_{N\times K}^{label}$ is the labeled data. For few-shot learning, the number of *N* could be small. For zero-shot learning, it corresponds to N<K. For incremental learning, it corresponds to N=N+j, where *j* is the number of new species discovered by the monitoring system.

*Few-shot/zero-shot inference.* Once the foundation models are trained they are used for inference. In our work, when the trained KI-CLIP is obtained on few-shot or zero-shot learning, the encoded human expert knowledge is fixed in the inference stage. The samples to be recognized are simply passed through the visual coder of the *pre-trained CLIP (#2 module)* in KI-CLIP and the similarity is then calculated with *T* to reach a conclusion. Since *T* is pre-calculated and stored during training, and the computations of the visual encoder and similarity are small, the computational overhead of KI-CLIP’s inference (i.e., recognition) is small enough that it can be performed without the need for dedicated accelerators. It also opens up the possibility of applying KI-CLIP to a wider range of embedded monitoring devices used on the fringe of the wildlife monitoring scene that are currently getting a lot of attention [3].

*Incremental learning.* The unseen and unknown species often encountered in the real world are dealt with using incremental learning in KI-CLIP. In practice, a coding expert’s knowledge *T*, that encompasses as many species as possible, will be constructed. Then, the value of I(·) is calculated according to Equation (5) and the incremental learning mechanism is triggered when I=1. In this case, on the one hand, we expect to use zero shots to identify unseen species as accurately as possible (i.e., when I=0 and the most stringent few-shot inference can be seen). Incremental learning is only triggered when the model is uncertain about its judgment. On the other hand, for unknown species we want to trigger incremental learning only if the model is not sure that it belongs to an existing species, in which case human expert knowledge is introduced. In this way, the KI-CLIP model will become increasingly accurate through the interactive intervention of human experts.

## 4. Experiments and Results

In this section, the experimental environments employed to assess the KI-CLIP method are first introduced. Then, three different types of experiments are conducted to validate the efficacy of the KI-CLIP method and the results are analyzed in the following sections. Particularly, the comparison experiments intend to assess the performance of KI-CLIP by comparing few-shot and zero-shot learning baseline methods over multiple datasets. The ablation experiments aim to analyze the effects of various components and hyperparameters in KI-CLIP on performance. The case studies seek to intuitively and vividly demonstrate the recognition results of KI-CLIP on a real wildlife dataset.

### 4.1. Experiment Environments

Since KI-CLIP has the advantage of requiring few training resources, we performed both the training and inference on a CPU. However, we also tested other baseline deep models in GPU environments. In our experiments, we used a *12th Gen Intel(R) Core(TM) i5-12400 2.50 GHz* CPU (*FP32 557 GFLOPS*) and an *NVIDIA Tesla T4* GPU (*FP32 8.1 TFLOPS*).

### 4.2. Experimental Comparison of Model Frameworks

#### 4.2.1. Few-Shot Leaning Analysis

Four few-shot baseline models, namely, CLIP, CLIP-Adapter, CoOp [85], and Tip-Adapter [86], were chosen to assess the capability of KI-CLIP to identify endangered and rare wildlife. Since previous studies [31,85,86,87] have demonstrated that CLIP possesses some capability for few-shot and zero-shot learning, but that neither is adequate for practical applications, zero-CLIP was employed as the baseline. The detailed settings are presented in Table 4.

**Figure 4:**
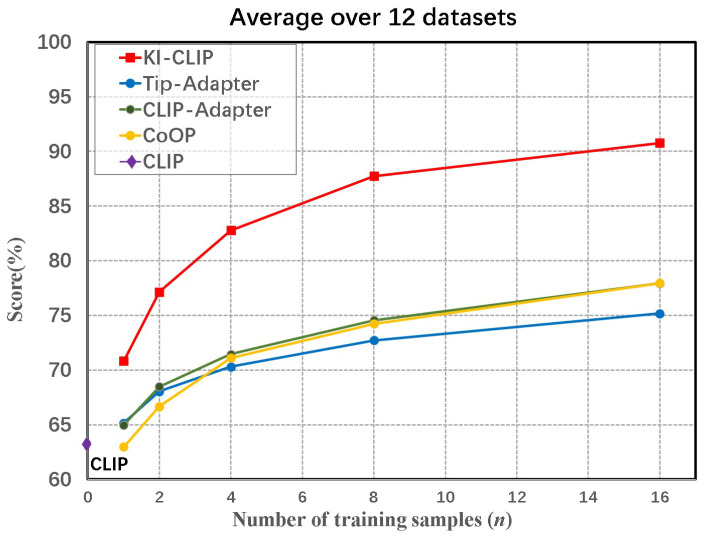
The average accuracy over 12 datasets with different numbers of training samples (*n*). KI-CLIP shows better performance than previous baselines across different few-shot setups.

**Figure 5:**
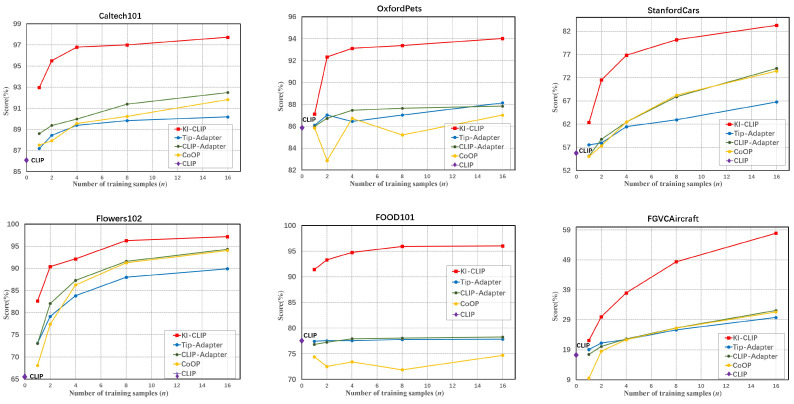

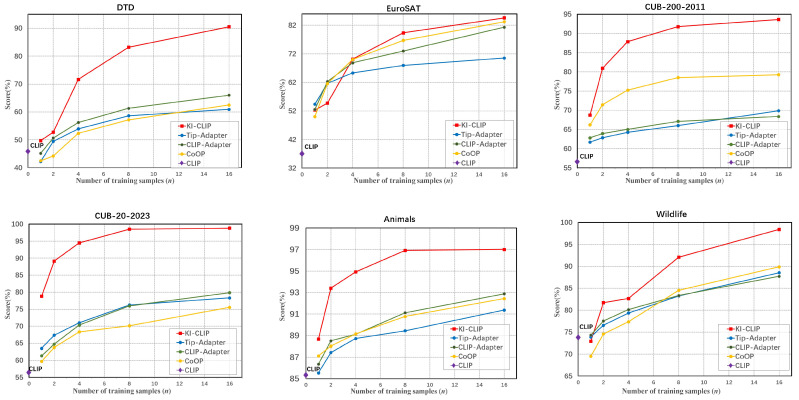
The results of few-shot learning with different numbers of training samples (*n*) on 12 datasets.

The main results of the few-shot learning are presented in Figure 4 and Figure 5 and Table 5. According to the average accuracy across all the 12 datasets shown in Figure 4, KI-CLIP clearly outperforms the other four baselines on different training sample settings, demonstrating the superior few-shot learning capacity of KI-CLIP. In addition, according to Figure 4, the accuracy of CLIP is only 63.35%, while various types of few-shot methods can significantly improve the accuracy in the case of small training samples. This shows that the data barrier faced in intelligent wildlife monitoring is indeed one of the challenges for applying large foundation deep models. Meanwhile, the few-shot baselines in Figure 4 achieve accuracies between 63% and 77% and they require 16 samples to achieve the best performance, while KI-CLIP requires only two samples to surpass them. This also means that in the field of wildlife monitoring it is not possible to use existing methods directly, but adaptations have to be made (as performed here with KI-CLIP).

We can also see from Figure 5 that KI-CLIP outperforms all baselines on each dataset. KI-CLIP is significantly ahead of the others on most datasets, especially on the animal related datasets. Figure 5 illustrates that, particularly for real wildlife data, KI-CLIP needs only 8 wildlife images to achieve over 90% accuracy, and 16 images to achieve over 97% accuracy. The accuracy is much higher than those previously reported for classical deep learning models (e.g., 88.9%) in a previous study [48]. These results demonstrate the competence of large foundation depth models to detect wildlife with little data, which is often the case for endangered and rare wildlife. Similarly, as shown in Table 5, the average accuracy of KI-CLIP is 90.76%, which is up to 20.72% better than the other few-shot models and 43.27% better than zero-CLIP. Such a high level of accuracy is sufficient for use in real-world wildlife monitoring systems. The results suggest that KI-CLIP is robust and can work well to detect endangered and rare wildlife beyond the data barrier in real field environments.

#### 4.2.2. Incremental Leaning Experiments Analysis

The incremental learning experiments focus on testing the ability of KI-CLIP to recognize unseen and unknown species. In this study, we conducted experiments on unseen learning and unknown learning.

*Analysis of unseen (zero-shot) learning.* The identification of unseen species can be demonstrated by the performance on zero-shot tasks. Therefore, $FI_{n}=0$ denotes the recognition of wildlife that has not been seen before. As CLIP can operate without training samples, it was chosen as the baseline model for the zero-shot task. For the purpose of describing wildlife monitoring applications, we conduct detailed comparative experiments on the *CUB-20-2023*, *Animals*, and *Wildlife* datasets to provide practical insights. In contrast to the few-shot and normal zero-shot experiments, we utilize a distinct evaluation criterion called *total accuracy* (TA). This involves testing the accuracy of the known species on a test sample (rather than a training sample), that is, identifying all unseen species. Specifically, TA counts unseen test samples as correctly identified if the monitoring methods discriminate them as any unseen species. TA was developed to simulate the actual working conditions of a wildlife monitoring system. Therefore, TA demonstrates the practical working capability of a wildlife monitoring system using our monitoring algorithm.

The results of the unseen (zero-shot) experiments are presented in Table 6. As shown in Table 6, the proposed KI-CLIP performs well in real-world scenarios with varying degrees of unseen incremental tasks, with a total accuracy of up to 95.58%, up to 29.79% more accurate than the baseline model. We also see from Table 6 that as the number of unseen species increases, the performance of KI-CLIP shows a decreasing trend when solving the more difficult problems, but the performance is still much higher than that of the baseline. At the same time, the minimum accuracy in this case is more than 83.54%, which can be well applied in real scenarios. In particular, the KI-CLIP method can achieve at least 94.46% and 91.25% total accuracy on the more species-rich Animals dataset and the real endangered and rare wildlife dataset despite the higher occurrence of unseen species (50% incremental degree). The results demonstrate that our proposed method KI-CLIP has good zero-shot learning capability and can monitor unseen endangered and rare wildlife well enough to meet the needs of practical applications.

*Analysis of unknown learning.* Unknown species tasks require KI-CLIP to be retrained. Therefore, the performance in this area is affected by the few-shot capability of KI-CLIP. Consequently, the ability of the model to detect uncertain targets is crucial to validate the success of the incremental learning mechanism according to Equation (5). Hence, we evaluated the classification probability values of the softmax classifier when classifying unfamiliar species and verified its incremental learning mechanism’s ability to deceive KI-CLIP. Similar to the unseen task, we used the *CUB-20-2023*, *Animal*, and *Wildlife* datasets from the animal category, as shown in Figure 1, for testing.

**Figure 6:**
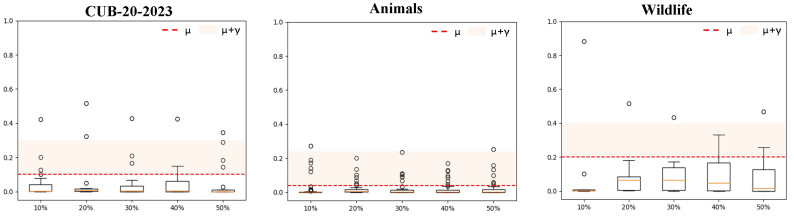
Box plots of the probability statistics of the *softmax* classifer on three animal datasets on unknown species recognition task. The horizontal coordinate is the incremental rate and the vertical coordinate is the accuracy. μ is the confidence level, and it is used as an indicator in the unknown task. The γ is 0.2 in this case.

Figure 6 shows box plots of the probability statistics of the *softmax* classifier. Figure 6 illustrates that KI-CLIP classifies unknown species with most of the identified classification probabilities being lower than μ according to Equation (5), and the medians are much smaller than the confidence level μ (i.e., $median_{CUB-20-2023}<\mu_{CUB-20-2023}$, $median_{Animals}<\mu_{Animals}$ and $median_{Wildlife}<\mu_{Wildlife}$). This means that the KI-CLIP method is confused about unknown species. At the same time, we can see in Figure 6 that there are a small number of outliers, indicating that the model has a relatively high confidence in classifying some unknown species. However, we can also see that these outliers are usually small, which means that we can adjust the incremental learning effect by the tolerance factor γ according to Equation (5). In this experiment, we eliminated more than 82.81% of the outliers by using a small tolerance coefficient (γ=0.2). The results suggest that the KI-CLIP method proposed in this paper can well perform incremental learning to improve the recognition accuracy of unknown species. Furthermore, the proposed KI-CLIP method can be made applicable to more diverse scenarios by simple weight adjustment.

#### 4.2.3. Computational Cost Comparison Analysis

Reduced computational cost is a vital aspect for KI-CLIP in order to cater to real-life intelligent wildlife monitoring requirements. Therefore, the training cost across the CPU and GPU, measured by TFLOPS per second, was compared to the baseline model while maintaining a certain level of performance. It is important to note that in order to demonstrate the superior performance of KI-CLIP with low computational resources (which allows us to learn incrementally), we used a strict or even biased experimental approach for KI-CLIP in these tests. Specifically, KI-CLIP was run only on a CPU environment while the other baseline models were run on GPUs. The mean accuracy across 12 datasets was used to assess the efficacy of both the few-shot and zero-shot methods.

Since the 16-shot task produces a performance that can fully satisfy real-world requirements, we compared the training costs of the 16-shot task and the results are shown in Table 7. As seen in Table 7, the computational cost of training CoOp and CLIP-Adapter is too high, taking up to 7 h. The results suggest that these two baselines cannot be applied to the unknown task in wildlife monitoring with incremental learning ability. Moreover, zero-CLIP requires no training and consumes no training resources. From a practical standpoint, obviously the low accuracy (63.35%) makes zero-CLIP useless. On the contrary, as shown in Table 7, KI-CLIP takes only 15.21 s to complete training and can achieve a practical level of accuracy (i.e., 90.75%). Although the Tip-Adapter has a slight time advantage (only 13.6 s of training time), the accuracy is too low (75.18% < 90.75%). Meanwhile, KI-CLIP can be trained by storing the encoded knowledge bank *T* so that the incremental training part takes only 0.56 s, which is much less than the baselines. Bringing different computing environments to the same level (i.e., average cost), the KI-CLIP method takes only 4.58 s to perform one TFLOPS of computation, which is several orders of magnitude lower than other methods. Taken together, KI-CLIP requires minimal training resources and can be retrained in a CPU environment. The low computational costs make the proposed KI-CLIP method more suitable for practical application scenarios in monitoring endangered and rare wildlife.

#### 4.2.4. Model Structure Analysis

As the experimental results have shown the advantages of the KI-CLIP, we briefly analyze the superiority of the structure of KI-CLIP by comparing our proposed KI-CLIP method with the related CLIP method and CLIP-Adapter [87] method.

*KI-CLIP vs. CLIP.* CLIP is a foundation deep learning model for multimodal fusion learning which is trained on a large amount of text and image data. CLIP shows the power in few-shot or even zero-shot learning [31] and it has been used successfully in medical [88], speech recognition [89], and other areas, but not in wildlife monitoring. In this work, we first introduce CLIP into intelligent wildlife as part of KI-CLIP. In this way, KI-CLIP also exploits the capabilities of multimodal fusion and few-shot/zero-shot learning. In contrast to the direct application of CLIP in various domains, KI-CLIP does not require the use of a certain number of samples to fine-tune CLIP. We train a lightweight neural network (i.e., SA-MLP) instead to help the CLIP in KI-CLIP work better by using fewer resources than fine-tuning. In addition, KI-CLIP combines human expert knowledge with the extensive image–text association knowledge of the pre-trained CLIP model. In short, KI-CLIP is a powerful large-scale deep learning model that is more applicable than CLIP to specific scenarios of endangered and rare wildlife monitoring.

**Figure 7:**
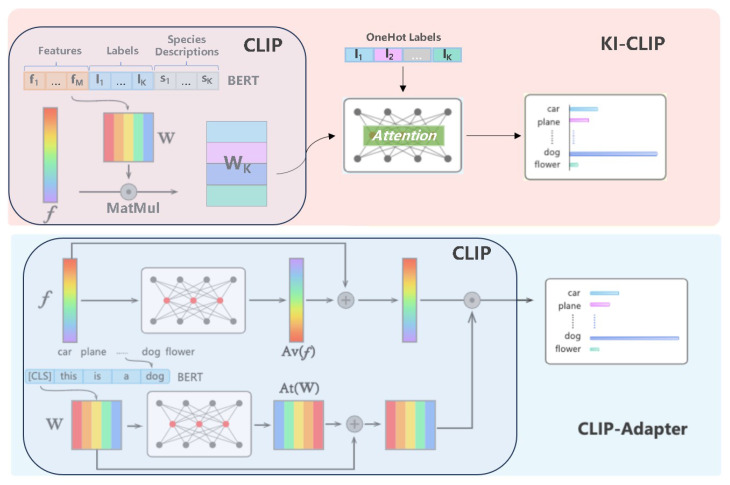
Illustration of crucial workflows of KI-CLIP and CLIP-Adapter.

*KI-CLIP vs. CLIP-Adapter.* CLIP-Adapter, which is similar to KI-CLIP, is a simple few-shot transfer method with extra inner layers [87]. Figure 7 shows the structures of KI-CLIP and CILP-Adapter. As shown in Figure 7, there are four distinct differences between KI-CLIP and CLIP-Adapter: (1) CLIP-Adapter is a variant of the CLIP method, i.e., it can become part of KI-CLIP by replacing the pre-trained CLIP module if necessary. (2) CLIP-Adapter still uses transfer learning for training, i.e., it requires the same amount of samples and computational resources as CLIP. As has been discussed many times, this approach is not suitable for monitoring endangered and rare wildlife. (3) KI-CLIP enables the wildlife monitoring system to have an incremental learning mechanism with the fixed CLIP modules and additionally the lightweight SA-MLP, which can be better applied to the monitoring of unseen and unknown species in the monitoring of endangered and rare wildlife (as discussed in detail in Section 3.2.1 and Section 3.2.2). (4) In contrast to CLIP-Adapter, the self-attention mechanism in KI-CLIP allows the model to focus on relevant expert knowledge from different fields. Furthermore, the self-attention and residual mechanisms in KI-CLIP reduce the computational costs.

In summary, the advantages of the architecture have been experimentally verified on 12 datasets where it significantly outperforms the current state-of-the-art few-shot methods. The theoretical comparison of the structure between KI-CLIP and the related methods also shows the superiority and wide applicability of KI-CLIP. Comparison experiments on four SOTA baseline models indicate that the KI-CLIP method achieves over 97% accuracy. Moreover, it improves the accuracy by up to 43.27%, with only 16 training samples at the second-level on a CPU, leading to a very low computational cost. If the accuracy is not essential, then only eight training samples are sufficient to exceed 90% accuracy. The results show that KI-CLIP can be used in real-world scenarios.

### 4.3. Ablation Analysis

Ablation experiments were used to determine the functions of the key components in KI-CLIP and the effects of the hyperparameters. According to the results of the comparison experiments (see Section 4.2) all methods performs best on the 16-shot task. To be fair, the 16-shot task was chosen as the experimental task for the ablation experiments.

#### 4.3.1. SA-MLP Analysis

As the pre-trained CLIP is fixed in KI-CLIP, the success of KI-CLIP relies heavily on the SA-MLP. Ablation experiments were conducted to assess the impact of the SA-MLP structure on the recognition performance. First, experiments were conducted to investigate the impact of varying quantities of hidden dense layers (denoted as *ablation exp 1*) and then the diverse quantities of nodes in the hidden layers and self-attention layer branches on the best x-shot task were investigated (denoted as *ablation exp 2*). Furthermore, to confirm the effect of the self-attention layer on the wildlife task the self-attention layer and residual structure were removed to examine the impact of the structure on the accuracy by administering the x-shot task on the animal datasets (denoted as *ablation exp 3*). Finally, we explored the role of the self-attention layer by comparing the training process with and without it (denoted as *ablation exp 4*). In order to conserve experimental resources, we chose four animal datasets and two random datasets (namely, DTD and FGVCAircraft) that showed satisfactory validation. In these experiments, *ablation exp 1* and *ablation exp 2* were performed on the selected six datasets to extensively validate the impact of the structure. While *ablation exp 3* was conducted on the four animal datasets, *ablation exp 4* was only carried out on the real Wildlife dataset due to limited experimental resources.

*The results of ablation exp 1.* Table 8 shows the accuracy of KI-CLIP with different numbers of hidden layers. As shown in Table 8, two hidden layers is the best structure of SA-MLP, performing best on all test datasets. A structure will not perform well if it is too shallow or too deep. From the machine learning view, the size of the data should match the ability of the model to learn, otherwise there will be overfitting or underfitting problems [90]. In our endangered and rare animal monitoring application, the data size is small, so that deeper models lead to underfitting and shallower models tend to overfitting. The result is also consistent with Table 8, where the accuracy is poor with one hidden layer and the accuracy increases with two layers but then decreases with the addition of new layers. The experimental results confirm the effectiveness of our SA-MLP lightweight design.

**Figure 8:**
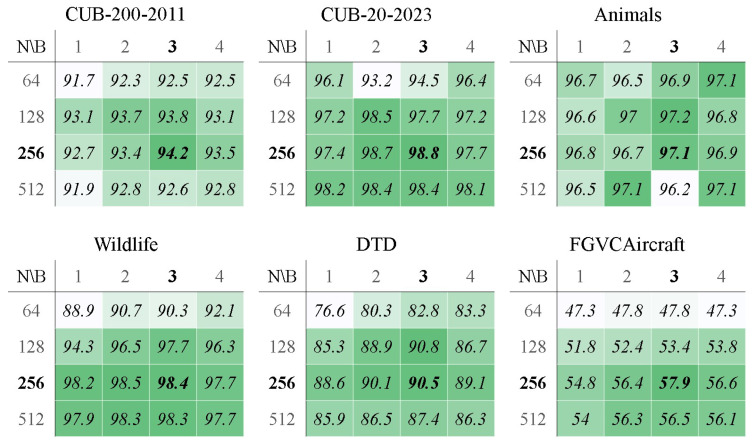
Heat maps of different structures of SA-MLP on 6 datasets with 16-shot task. N is the number of hidden layers and B is the size of the attention branches. The optimal network structure can be verified by testing different N and B combinations.

*The results of ablation exp 2.* Figure 8 shows the accuracy with different settings of the self-attention layers. Figure 8 illustrates that three attention branches with 256 hidden nodes is the best structure, as it gives the highest accuracy for most datasets. On the one hand, 256 nodes means that the pre-trained CLIP output feature dimension is 256 dimensions. An appropriate dimension size implies a trade-off between computational resources and performance. The experimental results show that 256 is an appropriate feature dimension size in the lightweight design used in this study. On the other hand, the experimental results show that KI-CLIP achieves the best performance when three branches are taken (as illustrated in Figure 8). This implicitly suggests that the three domains are related in expert knowledge. In conclusion, the results of *exp 1* and *exp 2* are evidence for the validity of the SA-MLP structure in KI-CLIP (illustrated in Figure 3).

*The results of ablation exp 3.* The results of ablation studies on components of SA-MLP are presented in Table 9. From Table 9, it can be seen that SA-MLP with the self-attention and residual modules has the highest recognition performance, with a minimum accuracy of 93.63% and a maximum of 98.41% on the Animals dataset, which can fully meet the actual monitoring requirements. Removing either of these two modules results in a rapid drop in accuracy. Removing the residual module reduces the average accuracy by 5.26%. When the self-attention module is removed, the average accuracy drops significantly, by 10.06%. Moreover, compared to the original MLP (without the self-attention mechanism and residual structure) the addition of the residual structure can improve the accuracy by about 3% on average, while the addition of the self-attention mechanism can improve the accuracy by 5% to 15%. This suggests that the self-attention mechanism and residual structure can effectively avoid overfitting in shallow networks with limited model structure capabilities. The experimental results demonstrate the effectiveness of the self-attention mechanism and the residual structure in SA-MLP.

**Figure 9:**
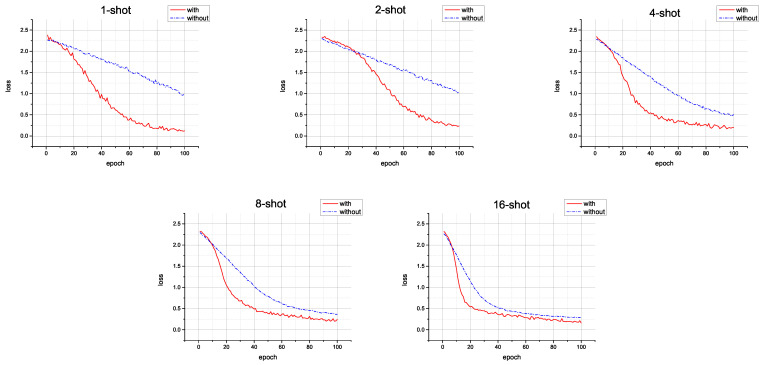
The training loss with/without self-attention layer on *Wildlife* dataset.

*The results of ablation exp 4.* The training losses with and without self-attention on different x-shot tasks are shown in Figure 9. As illustrated in Figure 9, self-attention significantly increases the ability of the KI-CLIP method to converge faster and reach lower values. This difference becomes even more significant as the size of the training sample becomes smaller. A fast-converging loss reduces the risk of overfitting in few-shot learning, helps achieve incremental learning with fewer resources, and indicates that self-attention could be efficient in real wildlife monitoring scenarios.

#### 4.3.2. Hyperparameters Analysis

As the selection of hyperparameters may depend on domain knowledge, we conducted experiments with various hyperparameters, such as visual encoder and learning rate, to determine their impact on the accuracy of the four animal datasets. Initially, we compared different visual encoders such as ViT-B/16, ViT-B/32, ResNet-50 and ResNet-101 used in KI-CLIP to demonstrate the advantages of our choice (i.e., ViT-B/16) in the present study. Then, various learning rates (lr) were applied. The experiments were carried out on the four animal datasets.

Table 10 shows the average accuracies of the different visual encoders ResNet-50, ResNet-101, ViT-B/16, and ViT-B/32 on the 16-shot task with the four animal datasets. First of all, similar to the comparison experiments, the results again demonstrate the superiority of our proposed KI-CLIP method in wildlife monitoring, as it achieves the best accuracy regardless of the visual encoder used, with a worst accuracy of 87.12% which is much better than the other baselines (at least more than 13.42%). In addition, KI-CLIP incorporates human expert knowledge where the other baselines do not. The results again show that this difference leads to a large performance improvement, which also implies the effectiveness of expert knowledge. Secondly, we see from Table 10 that as the coding model becomes larger and larger (ResNet-50 < ResNet-101 < ViT), better results can be achieved by all methods. This result also confirms the effect of introducing large foundation deep learning models. Finally, a smaller patch size leads to a performance improvement of more than 5%, suggesting the importance of fine-grained features in animal vision in the field of wildlife monitoring. It also implicitly refers to expert knowledge which typically characterizes a particular part of the animal. Hence, we choose the ViT-B/16 as the visual encoder in KI-CLIP.

**Figure 10:**
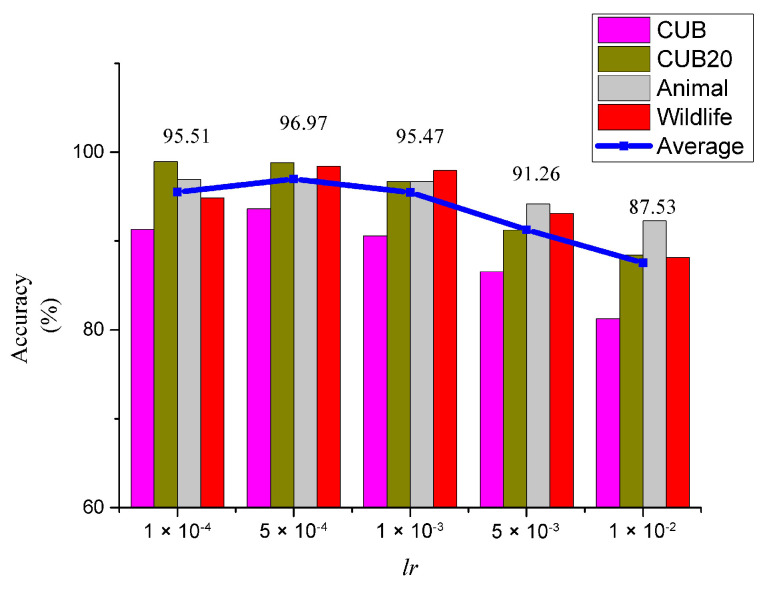
The accuracy on four animal datasets with different lr’s.

We then also tested the effect of different *lr*’s on the accuracy of the KI-CLIP method and the results are shown in Figure 10. KI-CLIP can achieve the highest average accuracy of 96.97% when the *lr* is 0.0005. The choice of different *lr*’s has an effect on the accuracy of the KI-CLIP method, as an *lr* that is too large drastically reduces the algorithmic precision. However, as long as the value of lr is not too large, the KI-CLIP accuracy does not differ by more than 2%, indicating that the KI-CLIP method is robust to this hyperparameter. The results suggest that a loose hyperparameter lr setting strategy can help KI-CLIP to adapt quickly to frequent incremental training in endangered and rare wildlife monitoring.

### 4.4. Case Study

In the case study, the performance of KI-CLIP was analyzed on real data containing rare and endangered wildlife. To demonstrate the effectiveness of KI-CLIP in accurately identifying wildlife, visualizations of the class weights assigned by *softmax* are provided and the training process of KI-CLIP in this weight classification is illustrated (denoted as *case exp 1*). Additionally, heat maps on the link between textual expert knowledge and images are drawn to demonstrate the successful utilization of knowledge from human experts (denoted as *case exp 2*). Thirdly, visualization techniques are used to directly present the incremental learning capability of KI-CLIP concerning the recognition effect of unfamiliar categories (denoted as *case exp 3*). Finally, we also tested the recognition accuracy of each individual species on the 16-shot task, since zoologists are sometimes concerned with a specific species when performing the unseen and unknown tasks with KI-CLIP (denoted as *case exp 4*). Since the case studies are intended to illustrate how KI-CLIP performs in real-world situations, we used the *Wildlife* dataset for all four experiments.

**Figure 11:**
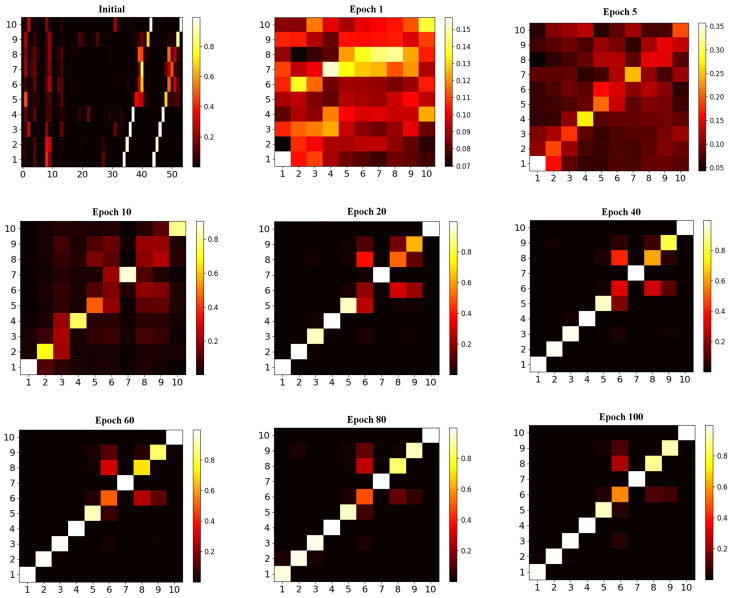
The weights of softmax during the training process on the *Wildlife* dataset. The horizontal coordinate in each subplot is the probability of the predicted category and the vertical coordinate is the category in which the training sample is located.

*The results of case exp 1.* The probability of softmax in different training stages on the *Wildlife* dataset are shown in Figure 11. Figure 11 provides an intuitive visualization of the process of rapid convergence of the KI-CLIP method. The softmax classification probability from the initial random state to 10 iterations can roughly classify all types correctly, and after dozens of iterations the probability on the diagonal line is the highest (the confidence is over 0.8). This indicates that a high level of accuracy has been achieved. This is similar to the phenomenon shown in Figure 9, that KI-CLIP has the ability to converge quickly. The results suggest that KI-CLIP can be quickly trained to obtain accurate recognition, which is useful in incremental learning.

**Figure 12:**
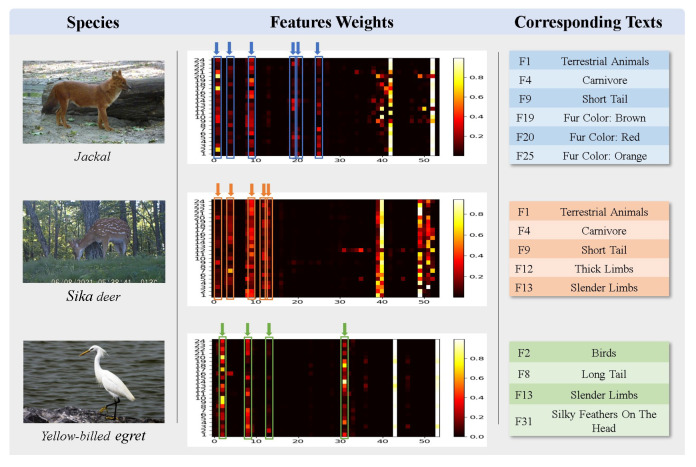
Heat maps for the *Wildlife* dataset. The heat maps were plotted according to the magnitude of the weights encoded by the fixed CLIP model. For simplicity, only the mapping relationships of the feature dimensions are drawn.

*The results of case exp 2.* Figure 12 shows a heat map of the association between test sample images and expert knowledge. As can be seen in Figure 12, the images are indeed associated with expert knowledge after fixed CLIP coding. For example, the regions with high linkage weights for yellow-billed egret contain features such as birds, long tail, etc. This demonstrates the excellent modeling ability of the underlying deep model to effectively use expert knowledge, allowing KI-CLIP to work effectively with only a few samples (i.e., few-shot learning tasks).

**Figure 13:**
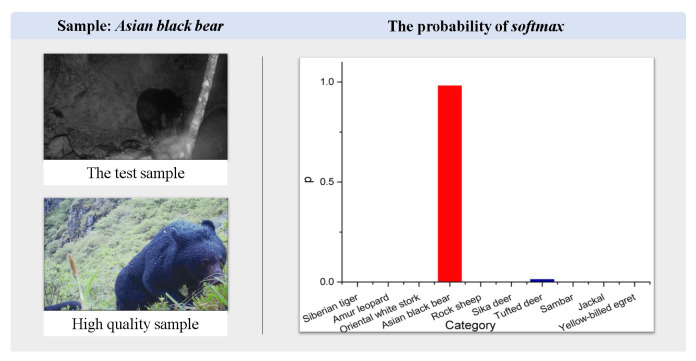
The correct recognition case on *Wildlife* dataset. The *Asian bear* is correctly classified.

**Figure 14:**
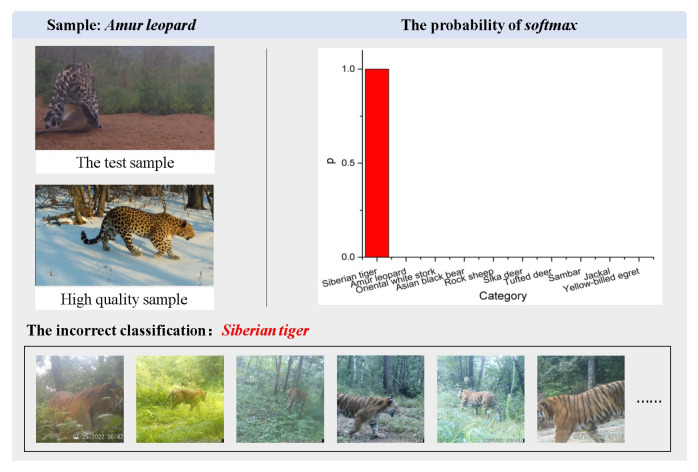
The incorrect recognition case on the *Wildlife* dataset. The *Amur leopard* photographed at specific viewpoints is mistaken for a *Siberian tiger*.

*The results of case exp 3.* To illustrate the ability of KI-CLIP to recognize unfamiliar species, we selected three typical recognition cases and used visual presentations. For the unseen task, classification accuracy was illustrated in the comparison and ablation experiments and we selected one correctly classified and one incorrectly classified case from the real dataset *Wildlife* for illustration. In the correct case, the *Asiatic black bear*, a grade II protected animal in China, was used as a test sample. As shown in Figure 13, a black bear in the night is correctly classified. We believe that the model-embedded expert knowledge “black bears” plays the biggest role in classifying animals of similar size. In the incorrect case, as shown in Figure 14, KI-CLIP incorrectly identified an *Amur leopard* test sample, photographed from behind with poor quality, as a *Siberian tiger*. From a human perspective, the animal in the test sample was the same size as the Siberian tiger and had the same patterned body, which was not easily discernible from the camera angle. Similarly, the Siberian tiger, apart from having a different pattern to the Amur leopard, also matches these characteristics when viewed from the rear. This may be the reason why KI-CLIP misclassified an Amur leopard as a Siberian tiger. The results suggest that, on the one hand, KI-CLIP has high recognition accuracy and can correctly classify animals based on most features. But, on the other hand, they also indicate that expert knowledge has a large impact on recognition.

**Figure 15:**
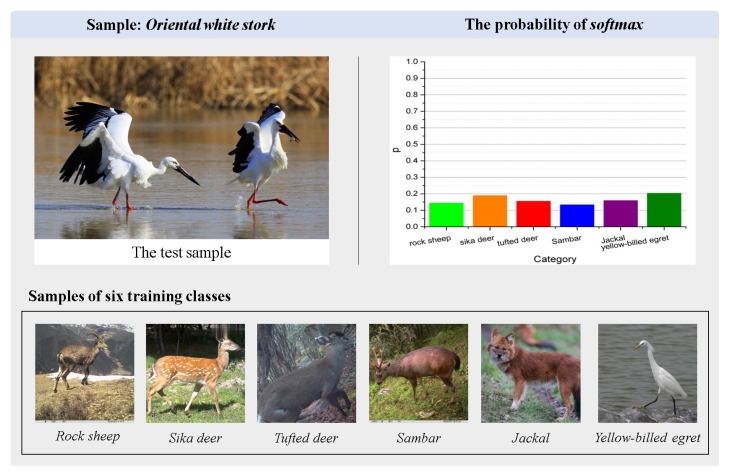
The unknown recognition case on the *Wildlife* dataset.

For the unknown task, the globally endangered *oriental white stork* was used as the unknown case. Among the known training species there are some that do not resemble the oriental white stork, as well as the *yellow-billed egret*, which is very similar to the oriental white stork. The yellow-billed egret is similar in size and appearance to the oriental white stork, but the expert description of “**bright yellow beak, and white plumage” still allows the general public to tell them apart. As can be seen from Figure 15, KI-CLIP does not classify the oriental white stork into the most similar yellow-billed egret among the six classes of training samples based on body size, wings, and other characteristics, but rather shows a clear state of confusion (i.e., the classification probability is low in all classes). Since the KI-CLIP has not seen the ongoing oriental white stork training samples before, the oriental white stork can be labeled as an unknown class under the KI-CLIP incremental learning mechanism. These results demonstrate the ability of KI-CLIP to discover new species.

**Figure 16:**
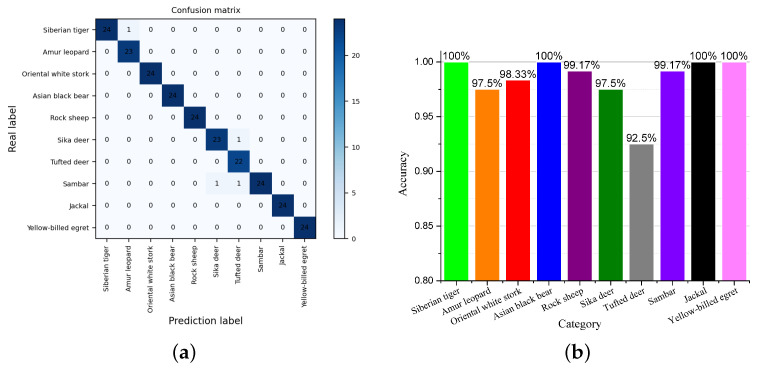
The breakdown of performance on individual species on the *Wildlife* dataset in the 16-shot task. (**a**) Confusion matrix of each species; (**b**) accuracy of each species.

*The results of case exp 4.* Figure 16 illustrates the breakdown of performance on individual species. Figure 16a shows the confusion matrix of the predicted categories and real labels. As can be seen from Figure 16a, most of the samples were correctly assigned to the correct label. A small number of samples were assigned under the wrong labels. For example, an *Amur leopard* sample was predicted to be a *Siberian tiger* and a *sika deer* sample was predicted to be a *sambar*. These very few incorrectly assigned samples were basically cases of poor sample quality with similar species (as described in *case exp 4*). As can be seen from Figure 16b, KI-CLIP can recognize *tufted deer* with the lowest accuracy in the few-shot task, but it also exceeds 92.5%. Moreover, KI-CLIP can recognize four species, namely, *Siberian tiger*, *Asian black bear*, *jackal*, and *yellow-billed egret*, with the highest recognition (accuracy = 100%). These results show that KI-CLIP can accurately recognize most species in real-world scenarios.

## 5. Discussion

In this work, we have investigated the solution that is the proposed KI-CLIP, using foundation deep learning models, to monitor endangered and rare wildlife in practice with few data and limited computation cost. First, the results show that the KI-CLIP method achieves over 97% accuracy in real-world monitoring of endangered and rare wildlife using only 16 samples, and KI-CLIP improves the recognition accuracy by up to 43.27% over other few-shot models on all 12 datasets. To date, the incremental intelligent monitoring algorithms only achieve 77.09% accuracy with less computational resources for recognition [55], which is only similar to the average accuracy of the few-shot baseline models in this paper. Furthermore, for some ideal algorithm case studies, although the best accuracy is close to 90% (about 88%, at least 9% lower than KI-CLIP) [38,48], they do not have the ability to discover new species. Although there have been some studies on wildlife monitoring [41], they are not only less accurate than KI-CLIP, but also require hundreds of training samples and cannot be directly applied to monitoring endangered and rare wildlife. However, our method requires only 8 or 16 samples to achieve impressive performance in practice and has great potential for application.

Then, by summarizing previous research we found that although intelligent algorithms in the field of wildlife monitoring use deep learning models such as VGG, CNN, ResNet-50, and GoogleNet [38,48], their capabilities are still dwarfed by those of the foundation models currently emerging, such as chatGPT and CLIP, which is also demonstrated by the ablation experiments in this work. In addition, the fine-tuning approach to train these models, which requires a certain amount of high-quality data, prevents them from being directly applied to the monitoring of endangered and rare animals. KI-CLIP enables the use of foundation deep learning models by designing a lightweight self-adaptive network. To our best knowledge, this is the first study to introduce foundation deep learning models such as CLIP to wildlife monitoring. Moreover, the CLIP model used in our work is fixed without training, allowing KI-CLIP to be trained on the CPU in seconds. This not only consumes very little computational resources but also gives it an incremental learning capability to effectively monitor undiscovered species.

Third, the few-shot results show that KI-CLIP is 16.46% better than CLIP-Adapter without expert knowledge (shown in Table 5). Despite the difference in structure between the two, it can be tentatively assumed that the difference in expert knowledge contributes to much of the improvement. In addition, the ablation experiments show that ViT encoders with different patch sizes lead to at least a 5% difference in performance and, together with the case study, textual expert knowledge, and wildlife image mapping experiments, it can be seen that expert knowledge does indeed establish a link with species’ fine-grained features in KI-CLIP. We believe that this adds an inductive bias [91] to the recognition model, increasing its robustness and generalization. Furthermore, as the textual human expert knowledge used in this paper is easily accessible, the strategies for utilizing specialist knowledge can be easily applied in practice.

Finally, the self-attention mechanism not only improves the detection performance of KI-CLIP but also allows the method to converge quickly with very few samples. This suggests that the self-attention mechanism not only avoids the occurrence of overfitting but also allows KI-CLIP to rapidly iterate under the incremental learning mode to detect unknown species.

To this end, the discovered empirical evidence indicates that the judicious introduction of the insights of specialist knowledge can significantly enhance the accuracy of the algorithm even with a small number of samples. The ablation experiments rigorously demonstrate the effectiveness and usefulness of the KI-CLIP components. In summary, the comprehensive experimental results demonstrate the accuracy of KI-CLIP in detecting wildlife using minimal resources and the ability to dynamically monitor emerging species. The findings indicate that KI-CLIP has the potential to be used for practical monitoring of rare and endangered wildlife. The main contributions are as follows:

- A new approach called KI-CLIP to monitor endangered and valuable wildlife is proposed. We introduce the power of large foundation deep learning models into wild animal protection for the first time (according to extensive research), with minimal data and computational resources.
- Human expert knowledge is successfully combined into a CLIP model for species recognition without training. The findings can lead to further research on intelligent monitoring of wildlife.
- In addition, a lightweight MLP recognition network, to further reduce training resources, which uses self-attention and residual mechanisms, is designed in KI-CLIP. The experimental results demonstrate its effectiveness.
- A probability-based incremental learning mechanism has been added to KI-CLIP to enable it to detect unmonitored and unknown species. At the same time, combined with KI-CLIP’s minimal training and inference resources, it allows our research to be applied to the practical monitoring of endangered and valuable wildlife.

A limitation of KI-CLIP is that it does not consider small target detection. Since aerial photography tools such as drones are widely used for wildlife monitoring, KI-CLIP would face challenges with small target detection. Although the highly accurate detection performance of KI-CLIP under the constraints of fewer samples and lower power consumption allows it to be carried out by drones, KI-CLIP is primarily designed for ground scenarios where animal targets are a large part of the population and its performance in detecting small targets is not necessarily very high. In future work, we will continue to optimize the small target detection performance of KI-CLIP for aerial photography scenarios.

## 6. Conclusions

This study is the first to explore a solution based on a foundation deep learning model for practical application in endangered and rare wildlife monitoring. In this study, we proposed a novel method called KI-CLIP to meet the requirements of high accuracy with few data, low computational cost, and incremental monitoring of unfamiliar species in practice. KI-CLIP introduces the powerful recognition capabilities of the foundation CLIP model and enables the foundation model to excel in endangered and rare wildlife monitoring scenarios where few-shot learning issues are encountered by incorporating expert knowledge. To solve another problem of computational cost in monitoring endangered and rare wildlife, KI-CLIP is designed with an additional lightweight adaptation network to fix the CLIP model without the need for training, significantly reducing the computational cost. In the adaptation network, the self-attention and residual module are added to avoid overfitting in such a simple network and improve the performance. In addition, KI-CLIP is designed with a very simple probability-based incremental learning module to solve the practical problem of encountering unfamiliar species during monitoring due to the very small amount of training resources required.

In order to accurately evaluate the performance of KI-CLIP in solving problems in monitoring endangered and rare wildlife we have conducted extensive experiments such as comparison experiments, ablation experiments, and case studies on 12 datasets of different types, including real monitoring data, animal data, and generic data. We have verified the excellent performance of KI-CLIP in the endangered and rare wildlife monitoring field and even in the generic few-shot learning field from different perspectives and with different tasks. With a recognition accuracy of over 97% on real data, the ability to train on the second stage of the CPU and the ability to discover new species, KI-CLIP is fully capable of real-world application scenarios for endangered and rare wildlife and can make new significant discoveries in the field of wildlife. To conclude, these results are significant because they are likely to be used for potentially important discoveries in the field of wildlife research.

## Figures and Tables

**Table 1:** Descriptions of the datasets.

No.	Name	Categories	Source	#Class	#Images	Image Size
1	Caltech101 [64]	General data	Benchmark datasets	101	9.1 k	300 × 200
2	OxfordPets [65]	General data	Benchmark datasets	37	7.4 k	224 × 224
3	StanfordCars [66]	General data	Benchmark datasets	196	16.2 k	224 × 224
4	Flowers102 [67]	General data	Benchmark datasets	102	8.2 k	224 × 224
5	Food101 [68]	General data	Benchmark datasets	101	101 k	512 × 512
6	FGVCAircraft [69]	General data	Benchmark datasets	10 k	9.1 k	600 × 600
7	DTD [70]	General data	Benchmark datasets	47	5.6 k	224 × 224
8	EuroSAT [71]	General data	Benchmark datasets	10	27 k	64 × 64
9	CUB-200-2011 [72]	Animal data	Benchmark datasets	200	11.8 k	600 × 600
10	CUB-20-2023	Animal data	Selected from benchmark datasets	20	1 k	600 × 600
11	Animals	Animal data	Selected from benchmark datasets	50	2 k	224 × 224
12	Wildlife	Endangered and rare wildlife data	Actual data from national parks in China	10	400	224 × 224

# represents the number.

**Table 2:** Details of the non-benchmark datasets used in the present work.

Dataset	Species	Examples
CUB-20-2023	Black-footed albatross, gray catbird, least auklet, rusty blackbird, bobolink, groove-billed ani, painted bunting, sooty albatross, brewer blackbird, indigo bunting, parakeet auklet, spotted catbird, cardinal, Laysan albatross, red winged blackbird, yellow breasted chat, crested auklet, lazuli bunting, rhinoceros auklet, yellow headed blackbird.	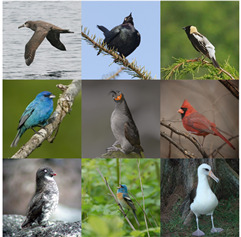
Animals	Alouatta, cheetah, hippo, noisy night monkey, sheep, antelope, chimpanzee, horse, otter, skunk, argali sheep, cow, jaguar, panda, snow leopard, beaver, deer, king cheetah, panther, spider monkey, bison, dog, leopard, pig, squirrel, black bear, elephant, lion, polar bear, Siberian tiger, bobcat, fox, marmot, rabbit, uakari, brown bear, giraffe, monkey, raccoon, weasel, buffalo, gorilla, moose, rat, wolf, cat, hamster, mouse, rhino, zebra.	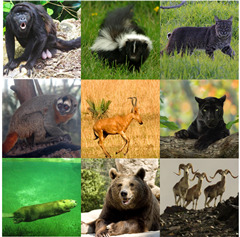
Wildlife	Siberian tiger, sika deer, Amur leopard, tufted deer, oriental white stork, sambar, Asian black bear, jackal, rock sheep, yellow-billed egret.	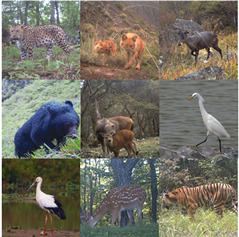

**Table 3:** Descriptions of specialist knowledge texts.

#	Field	Description
1	Features	The morphological characteristics recover the distinction between different species in animal taxonomy, such as coat color, body type, and so on.
2	Species Name	The names of species used in KI-CLIP.
3	Species Description	A short summary for each species of no more than 100 words.

**Table 4:** The experimental settings used in comparison experiments.

#	Configuration Item	Settings
1	Baseline Models	CLIP, CoOp, CLIP-Adapter, and Tip-Adapter.
2	Few-shot Settings	n=1,2,4,8,16
3	Training Settings	Datasets: 12 datasets shown in Figure 1; optimizer: Adam, lr=0.0005; max epochs: 200; visual encoder: ViT-B/16; other hyperparameters are set by default.
4	Inference Settings	Twenty percent of each dataset is selected as the test dataset. As the wildlife animal dataset is much smaller than the others, the same random size test split is used, but repeated execution is used for a fair comparison. The same number of test samples (24) is randomly selected from each species, in total 24×K test samples, and each test is repeated 5 times.
5	Metric	The mean accuracy of the repeat testing results.

**Table 5:** The average accuracy compared to the four baseline models on different few-shot setups.

Samples	KI-CLIP (Accuracy Value) *	Tip-Adapter	CLIP-Adapter	CoOp	CLIP
1	70.81%	62.10% (8.71%)	61.81% (9.00%)	58.34% (12.47%)	59.03% (11.78%)
2	77.15%	63.78% (13.37%)	64.51% (12.64%)	61.45% (15.70%)	55.37% (21.78%)
4	82.78%	65.05% (17.73%)	67.22% (15.56%)	66.35% (16.43%)	52.11% (30.67%)
8	87.74%	67.08% (20.66%)	70.05% (17.69%)	69.54% (18.20%)	49.23% (38.51%)
16	90.76%	70.04% (20.72%)	74.30% (16.46%)	74.34% (16.42%)	47.49% (43.27%)

* The values in brackets are the improvement of KI-CLIP.

**Table 6:** The total accuracy of KI-CLIP and the improvements compared to the baseline in the unseen task.

Incremental Rate	CUB-20-2023		Animals		Wildlife	
	*Improvement*	*KI-CLIP*	*Improvement*	*KI-CLIP*	*Improvement*	*KI-CLIP*
10%	+24.82%	93.57%	+5.58%	95.58%	+14.6%	95.42%
20%	+29.79%	89.17%	+6.7%	93.78%	+5.34%	92.16%
30%	+25.77%	88.96%	+4.92%	94.92%	+11.21%	93.38%
40%	+15.55%	86.38%	+1.13%	92.58%	+17.65%	94.32%
50%	+10.21%	83.54%	+5.96%	94.46%	+6.25%	91.25%

**Table 7:** The training cost results.

Method	X-Shot	Training	Epochs	Time	Accuracy (%)	Average Cost (s/TFLOPS)
CLIP	0	Free	0	0	63.35	0
Tip-Adapter	16	Training	0	**13.6 s**	75.18	**108.8**
CoOp	16	Training	200	7 h 20 min	77.95	1,056,000
CLIP-Adapter	16	Training	200	25 min	77.93	12,000
KI-CLIP	16	Training	200	15.21 s (**0.56 s** *)	**90.75**	121.68 (**4.58** *)

* represents the cost used by training part of model.

**Table 8:** The accuracy of KI-CLIP with different numbers of hidden layers.

Metric	1	2	3	4	5
CUB-200-2011	56.67	**93.63**	87.17	84.96	72.89
CUB-20-2023	86.45	**98.82**	97.19	95.25	94.32
Animals	93.21	**97.1**	96.31	93.25	85.88
Wildlife	86.25	**98.41**	96.58	95.12	92.65
DTD	59.39	**90.51**	84.46	78.74	40.69
FGVCAircraft	22.38	**57.92**	51.26	43.25	23.58

The unit of accuracy in this table is %, and the bold values are the best ones.

**Table 9:** The accuracy of component ablation studies on 16-shot task.

Method	Self-Attention	Residual	CUB-200-2011	CUB-20-2023	Animals	Wildlife	Mean
SA-MLP	✔	✔	**93.63**	**94.12**	**97.01**	**98.41**	**95.79**
SA-MLP	✔		91.72	86.45	95.33	88.65	90.54
SA-MLP		✔	77.36	87.08	90.83	87.66	85.73
SA-MLP			76.85	86.39	91.12	83.75	84.53

The unit of accuracy in this table %, and the bold values are the best ones.

**Table 10:** The average accuracy with different visual encoders on four animal datasets.

Methods	ResNet-50	ResNet-101	ViT-B/32	ViT-B/16
CLIP	69.94	67.74	68.88	72.42
CoOp	74.37	78.89	80.32	84.28
CLIP-Adapter	73.71	75.32	77.67	82.21
Tip-Adapter	72.43	75.12	76.89	81.91
**KI-CLIP**	**87.12**	**88.54**	**91.78**	**96.97**

The unit of accuracy in this table is %, and the bold values are the best ones.

## Data Availability

The data used to support the findings of this study are available from the corresponding author upon request.
